# The fungicide dodine primarily inhibits mitochondrial respiration in *Ustilago maydis*, but also affects plasma membrane integrity and endocytosis, which is not found in *Zymoseptoria tritici*^☆^

**DOI:** 10.1016/j.fgb.2020.103414

**Published:** 2020-09

**Authors:** Martin Schuster, Gero Steinberg

**Affiliations:** a School of Biosciences, University of Exeter, Exeter EX4 4QD, UK; b University of Utrecht, Padualaan 8, Utrecht 3584 CH, The Netherlands

**Keywords:** N-dodecyl guanidine acetate, Endocytosis, PM fluidity, Fungicide, Mode of action

## Abstract

Early reports in the fungus *Ustilago maydis* suggest that the amphipathic fungicide dodine disrupts the fungal plasma membrane (PM), thereby killing this corn smut pathogen. However, a recent study in the wheat pathogen *Zymoseptoria tritici* does not support such mode of action (MoA). Instead, dodine inhibits mitochondrial ATP-synthesis, both in *Z. tritici* and *U. maydis*. This casts doubt on an fungicidal activity of dodine at the PM. Here, we use a cell biological approach and investigate further the effect of dodine on the plasma membrane in both fungi. We show that dodine indeed breaks the integrity of the PM in *U. maydis*, indicated by a concentration-dependent cell depolarization. In addition, the fungicide reduces PM fluidity and arrests endocytosis by inhibiting the internalization of endocytic vesicles at the PM. This is likely due to impaired recruitment of the actin-crosslinker fimbrin to endocytic actin patches. However, quantitative data reveal that the effect on mitochondria represents the primary MoA in *U. maydis*. None of these plasma membrane-associated effects were found in dodine-treated *Z. tritici* cells. Thus, the physiological effect of an anti-fungal chemistry can differ between pathogens. This merits consideration when characterizing a given fungicide.

## Highlights

• The fungicide dodine inserts into the PM of the corn smut fungus *Ustilago maydis.*
• Dodine depolarises cells and reduces the PM fluidity in *U. maydis*, but not in *Z. tritici.*
• Dodine impairs actin patch motility in *U. maydis*, due to impaired recruitment of fimbrin, which arrests endocytosis*.*
• The MoA at the PM is secondary, as oxidative phosphorylation is inhibited at much lower dodine concentrations.
• Fungal pathogens can differ in their response to a given fungicide.

## 1 Introduction

The world’s food security is based on few calorie crops, including maize, wheat and rice, with these three crops alone providing two thirds of the calories in human diet (Cassman, 1999). Fungal crop pathogens pose the most serious threat to this supply (Fisher et al., 2012), and our best “weapon” in the protection of crops against fungal diseases are fungicides (Leadbeater, 2015, Oliver and Hewitt, 2014). In many cases the way fungicides kill the pathogen (the mode of action, MoA) is poorly understood. However, the burden of fungal disease requires up to 10 fungicide applications per season (e.g.https://thefruitgrower.co.uk/where-are-we-with-scab-control/). As this can expose both consumers and the environment to significant amounts of anti-fungal chemistries, a detailed understanding of their cellular activity is paramount (Oliver and Hewitt, 2014).

Multi-site fungicides, such as the protectant fungicide dodine (=Syllit C400), attack the pathogen by inhibiting more than one cellular process. However, dodine's MoA is not understood, and, consequently, the fungicide is listed in the FRAC code© list 2020 as having an “unknown MoA” (classification U12; https://www.frac.info). The amphipathic nature of dodine suggests that the molecule inserts into the fungal plasma membrane (PM). Indeed, investigation of the physiological effect of dodine in various fungi conclude that dodine disrupts the integrity of the fungal PM (Brown and Sisler, 1960, Kottke and Sisler, 1962, Miller and Barran, 1977, Yao et al., 1995). This activity increases the permeability of the cell and is thought to result in death of the pathogen, as was concluded in the maize pathogen *Ustilago maydis* (Solel and Siegel, 1984). However, other reports provide indications that dodine acts inside the fungal cell, where it appears to inhibit vital enzymes and cellular respiration (Brown and Sisler, 1960, Somers and Fisher, 1967, Somers and Pring, 1966).

Most recently, we followed a cell biological approach in *Zymoseptoria tritici* to better understand the MoA of dodine in fungal pathogens, which led to the discovery of a new antifungal chemistry (Steinberg et al., 2020). *Z. tritici* is a major pathogen on wheat in temperate climate, causing yield loss worth over £200 million in the UK per year (Fones et al., 2020). Despite this impact, the cellular biology of *Z. tritici* is poorly understood (Steinberg, 2015), which prompted us to develop molecular live cell imaging tools for cell biological studies (e.g. Guo et al., 2015, Kilaru et al., 2015, Kilaru et al., 2017, Schuster et al., 2015). We used these tools to assess the effect of dodine on living cells and found that the fungicide targets fungal mitochondria, where it interferes with NADH oxidases and depolarises the inner membrane. As a consequence, ATP synthesis is impaired, which eventually kills the pathogen cell (Steinberg et al., 2020). Surprisingly, these studies did not reveal a disruptive activity of dodine at the PM of *Z. tritici*, and thus add further doubt to a primary MoA at the PM of fungi, such as *U. maydis*.

Here, we exploit established live cell imaging tools and techniques for *U. maydis* (e.g. Steinberg and Schuster, 2011) and *Z. tritici* (Guo et al., 2015, Kilaru et al., 2015, Kilaru et al., 2017, Schuster et al., 2015) and compare the effect of dodine on the two fungi. Using sub-lethal concentrations of dodine, we identified early responses of the fungal cell to the presence of the fungicide. We report that dodine alters the phenotypic appearance of the PM in *U. maydis*. This is accompanied by an increase in ion permeability, resulting in membrane depolarisation. In addition, dodine decreases fluidity of the PM in *U. maydis*, indicated by fluorescent recovery after photo-bleaching (FRAP) experiments. Finally, the dynamics of PM-associated actin patches are impaired and endocytosis is inhibited. This is possibly due to abolished targeting of the cross-linking protein fimbrin to the site of endocytioc internalization. While dodine also alters the PM appearance in *Z. tritici*, none of these *U. maydis* phenotypes were found in this wheat pathogen. Thus, the two fungi react differently to dodine, suggesting that the physiological effect of a fungicide could vary between fungal pathogens.

## 2 Results and discussion

**Fig. 1:**
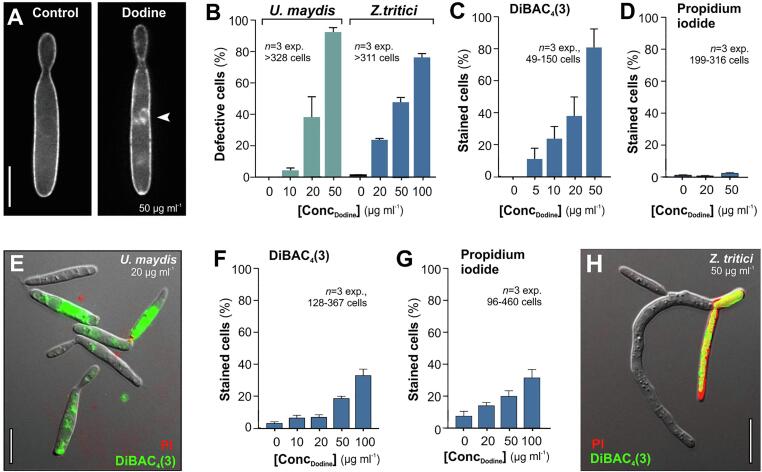
Effect of dodine on the PM in *U. maydis* and *Z. tritici.* (**A**) Appearance of the PM, labeled by the fluorescent syntaxin GFP-Sso1 (Steinberg and Schuster, 2011) in yeast-like cells of *U. maydis*. At higher concentrations, dodine induces peripheral accumulations of the membrane-bound marker protein. Note that (i) similar structures have been reported in *Z. tritici* (Steinberg et al., 2020) and (ii) these structures represent tubular invaginations of the PM (see Supplementary Fig. 1). The concentration of dodine used is indicated in the Figure; cells were treated for 30 min. Scale bar represents 5 µm. (**B**) Bar chart showing the number of *U. maydis* and *Z. tritici* cells with peripheral GFP-Sso1-positive invaginations at various concentrations of dodine. Bars show mean ± standard error of the mean (SEM). The samples size *n* is indicated in the graph. (**C**) Bar chart showing the number of *U. maydis* cells that stained with the membrane potential indicator DiBAC_4_(3) after 30 min treatment with various concentrations of dodine. Note that dodine depolarizes the cells. Bars show mean ± standard error of the mean (SEM). The samples size *n* is indicated in the graphs.(**D**) Bar chart showing the number of *U. maydis* cells that stained with the cell viability-reporter propidium iodide after 30 min treatment with various concentrations of dodine. While cells are depolarized, they do not take up propidium iodide, suggesting that dodine permeabilizes the PM for ions, before it kills the cell. Bars show mean ± standard error of the mean (SEM). The samples size *n* is indicated in the graphs. (**E**) Dodine-treated *U. maydis* cells, co-stained with DiBAC4(3) (green) and propidium iodide (red). The concentration of dodine used is indicated in the Figure; cells were treated for 30 min. Several cells are depolarized, indicated by the uptake of the voltage-sensitive dye DiBAC4(3), but have not yet taken up the viability reporter propidium iodide. Scale bar represents 10 µm. (**F**) Bar chart showing the number of *Z. tritici* cells, stained with DiBAC_4_(3) after 30 min treatment with various concentrations of dodine. Note that the voltage-sensitive dye enters some cells at high concentration. Bars show mean ± standard error of the mean (SEM). The samples size *n* is indicated in the graphs. (**G**) Bar chart showing the number of *Z. tritici* cells, stained with propidium iodide after 30 min treatment with various concentrations of dodine. Note that the number of stained cells is almost identical to the number of depolarized cells (see (**F**), suggesting that DiBAC_4_(3) entered the pathogen upon its death, which suggests that membrane depolarization is a secondary consequence of death-associated membrane permeabilization. Bars show mean ± standard error of the mean (SEM). The samples size *n* is indicated in the graphs. (**H**) Dodine-treated *Z. tritici* cells, co-stained with DiBAC_4_(3) (green) and propidium iodide (red). A dead cell also contains the voltage-sensitive dye. The concentration of dodine used is indicated in the Figure; cells were treated for 30 min. Scale bar represents 10 µm. (For interpretation of the references to colour in this figure legend, the reader is referred to the web version of this article.)

Dodine was reported to act on the PM of *U. maydis* (Solel and Siegel, 1984). We tested if such activity alters the appearance of the PM by visualizing the fluorescent PM syntaxin reporter GFP-Sso1 (Steinberg and Schuster, 2011). We applied dodine in concentrations up to 50 µg ml^−1^, for 30 min, and investigated the phenotypic appearance of the PM by epi-fluorescent microscopy. We found that high concentrations of dodine induce PM-associated GFP-Sso1 “patches” (Fig. 1A, 1B). Electron microscopy revealed extended PM invaginations in dodine-treated *U. maydis* cells (Supplementary Fig. 1). Inwards folding of the PM was previously reported in bacteria and is thought to be due to the insertion of the lipophilic cation into the membrane (Cabral, 1992). Dodine also causes peripheral GFP-Sso1-patches in *Z. tritici* (this study; Steinberg et al., 2020), but, in this fungus, this phenotype occurs at much higher concentrations of dodine (∼100 µg ml^−1^; Fig. 1B). This result provided first indication that the PM of both fungi interacts differently with the fungicide.

We next set out to investigate if dodine compromises the integrity of the PM by monitoring ion permeability in dodine-treated cells. Eukaryotic cells selectively pump ions across their PM, which generates an electric potential over the membrane (Shapiro, 2000). An increase of ion permeability depolarizes the cell, which can be monitored by visualizing cellular accumulation of the anionic voltage-sensitive green-fluorescent oxonol DiBAC_4_(3) (Shapiro, 2000, Yamada et al., 2001). We used this dye to test if dodine increases depolarization, and thus ion permeability, of the PM in *U. maydis* and *Z. tritici* cells. Indeed, we found that dodine raised the number of DiBAC_4_(3)-positive *U. maydis* cells (Fig. 1C), with an estimated 50% inhibition at EC_50_ = 26.5 µg ml^−1^; Supplementary Fig. 2). Staining with the vitality marker propidium iodide, which only enters dead cells (Crowley et al., 2016), revealed that under these conditions, *U. maydis* cells remain alive (Fig. 1D, 1E). These results suggest that dodine inserts into the PM of *U. maydis*, opening it up for uncontrolled ion passage through the membrane, before killing the pathogen. We next tested for such an effect of dodine on the integrity of the PM in *Z. tritici.* We found that dodine is more toxic to this wheat pathogen, indicated by the increase of propidium iodide-positive cells (Fig. 1G). However, DiBAC_4_(3) staining did not reveal an increase in stained cells (Fig. 1F), and even at 100 µg ml^−1^ the number of depolarized cells did not differ significantly from that of propidium iodide-positive cells (Student's *t*-test witch Welch's correction, P = 0.9921). Co-staining of propidium iodide and DiBAC_4_(3) in dodine-treated cells demonstrated that the voltage-sensitive dye is only entering dead cells (Fig. 1H; yellow staining results from the presence of both dyes in the same cell). We conclude that dodine-dependent ion permeabilization of the PM precedes cell death in *U. maydis*, but not in *Z. tritici*. Thus, the fungicide differently affects the integrity of the PM in these fungi.

**Fig. 2:**
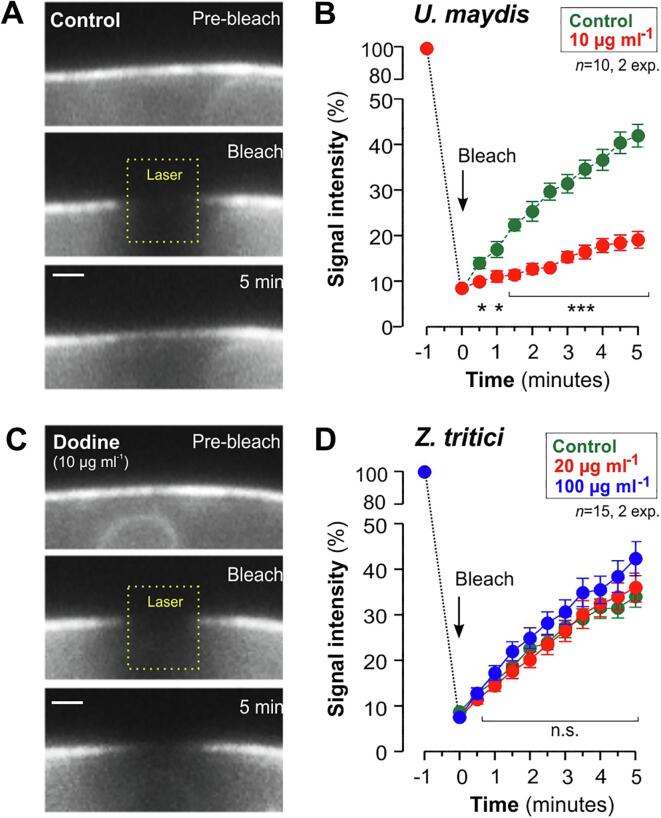
Effect of dodine treatment on the fluidity of the PM (**A**) Image series demonstrating fluorescent recovery of GFP-Sso1 in a photo-bleached area of the PM in a solvent-treated *U. maydis* cell (Control). The area of laser-bleaching is indicated by yellow box and “Laser”. Pre-bleach: image taken before photo-bleaching; Bleach: Image taken directly after laser photo-bleaching; 5 min: Image take after 5 min recovery time. The scale bar represents 1 µm. See also Video1. (**B**) Graphs showing GFP-Sso1 fluorescent recovery in a photo-bleached region of the PM of *U. maydis*. Data points represent mean ± standard error of the mean (SEM); samples size *n* is 10 from 2 experiments. All data sets were tested for normal distribution. All data sets which passed a Shapiro-Wilk test for normality (P > 0.05) a pairwise comparison of the two data sets at a given time point was done using a Student’s *t*-test with Welch’s correction, all data sets which did not passed a Shapiro-Wilk test for normality (P > 0.05) were compered using a nonparametric two-tailed Mann-Whitney test; single asterisk indicates significant difference at P < 0.05; triple asterisks indicates significant difference at P < 0.0001. See also Video1. (**C**) Image series demonstrating fluorescent recovery of GFP-Sso1 in a photo-bleached area of the PM in a *U. maydis* cell, treated with 10 µg ml^−1^ dodine (Dodine). The area of laser-bleaching is indicated by yellow box and “Laser”. Pre-bleach: image taken before photo-bleaching; Bleach: Image taken directly after laser photo-bleaching; 5 min: Image take after 5 min recovery time. Note that fluorescent recovery is slower than in (**A**). The scale bar represents 1 µm. (**D**) Graphs showing GFP-Sso1 fluorescent recovery in a photo-bleached region of the PM of *Z. tritici*. Data points represent mean ± standard error of the mean (SEM); samples size *n* is 15 from 2 experiments. All data sets were tested for normal distribution. On all data sets which passed the Shapiro-Wilk test for normality (P > 0.05) the comparison of 3 datasets at a given time was done using one-sided ANOVA test. All data sets which did not passed a Shapiro-Wilk test for normality (P > 0.05) were compered using a Kruskal-Wallis test; n.s. indicates non-significant difference at P > 0.05; (For interpretation of the references to colour in this figure legend, the reader is referred to the web version of this article.)

The formation of membrane invaginations in fungi and bacteria strongly suggests that the lipophilic cation dodine inserts into the PM. To investigate if this change in membrane composition and alters the fluidity of the PM, we performed fluorescent recovery after photo-bleaching experiments (FRAP) in dodine-treated cells of *U. maydis* and *Z. tritici* that express the membrane-bound reporter GFP-Sso1. We photo-bleached the fluorescent marker and monitored the recovery of fluorescence due to lateral diffusion of unbleached GFP-Sso1. In control experiments, photo-bleached areas in the PM showed partial recovery of GFP fluorescence within several minutes (Fig. 2A, 2B; Video 1). However, even at low concentration of dodine (10 µg ml^−1^), recovery of fluorescence was significantly impaired (Fig. 2B, 2C; Video 1), suggesting that diffusion of GFP-Sso1 within the PM is restricted by the presence of the lipophilic cation. Interestingly, no such effect was seen in *Z. tritici* cells, and even 100 µg ml^−1^ dodine did not inhibit fluorescent recovery of GFP-Sso1 in photo-bleached regions of the PM (Fig. 2D). This result further support the notion that the fungicide dodine shows different effects on the PM in both fungi.

Fungi undergo endocytosis (Peñalva, 2010), which was shown in *U. maydis* to be crucial for cell morphogenesis (Wedlich-Söldner et al., 2000) and plant pathogenicity (Fuchs et al., 2006). Moreover, moving early endosomes perform numerous important functions in *U. maydis*, including the spatial distribution of other organelles (Guimaraes et al., 2015, Lin et al., 2016) and long-range signaling during plant infection (Bielska et al., 2014; overview in Steinberg et al., 2017a). Endocytosis begins at the PM, where lipid composition, membrane surface charge and fluidity appears to control the formation of endocytic vesicles and their uptake into the cytoplasm, both in fungi (Degreif et al., 2019, Munn et al., 1999) and in human cells (Ben-Dov and Korenstein, 2013). We show here that dodine treatment alters PM fluidity in *U. maydis*. We therefore set out to investigate if the fungicide inhibits early steps of endocytosis at the PM.

**Fig. 3:**
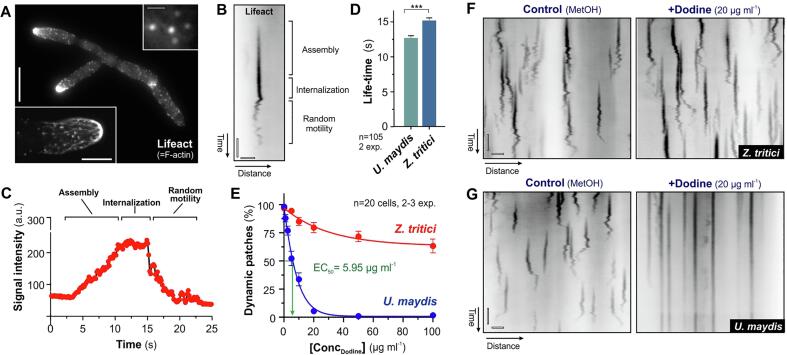
Effect of dodine on actin patch dynamics (**A**) The F-actin cytoskeleton in *Z. tritici*, visualized by labelling with the reporter Lifeact (Riedl et al., 2008), optimized for use in *Z. tritici* (Kilaru et al., 2017). F-actin cables concentrate at the growing tip (lower left insert), whereas actin patches are scattered at the periphery along the entire length of the cell (upper right insert). Note that the macropycnidiospore shown here consists of at least 4 cells. Sale bars: 10 µm (overview), 2 µm (lower left insert), 1 µm (upper right insert). See also Video 2. (**B**) Kymograph showing the dynamic behavior of an actin patches in *U. maydis* cell. The actin patch was labelled using Lifeact. Three different phases of behavior are indicated (for description see **C**). Horizontal bar: 1 µm, vertical bar: 3 s. (**C**) Graph showing Lifeact fluorescent intensity in an actin patch over time. In the “Assembly” phase, the signal intensity increases, until a plateau is reached. During this phase (“Internalization“), the endocytic vesicle gets formed. After scission of the vesicle, the Lifeact signal intensity rapidly decreases and shows random motility (“Random motility”). Intensities were taken from kymograph shown in (**B**). See also Video 3. (**D**) Bar chart showing the average ”life-time“ of actin patches in *U. maydis* and *Z. tritici*. Bars represent mean ± standard error of the mean (SEM); the samples size *n* is indicated in the graph. Data sets pass Shapiro-Wilk normality test (P > 0.05); pairwise comparison was done using a Student’s *t*-test with Welch’s correction, with triple asterisk indicating significant difference at P < 0.0001. (**E**) Graph showing the effect of various concentrations of dodine on actin patch motility in *U. maydis* and *Z. tritici*. Cells were incubated for 30 min. Data points are given as mean ± standard error of the mean (SEM) from 20 cells and 2–3 experiments. Concentration of dodine at 50% inhibition of patch motility is indicated by EC_50_ value (determined graphically, green arrow). (**F**) Kymographs showing the dynamic behavior of actin patches in *Z. tritici*, treated with the solvent methanol (Control) and with 20 µg ml^−1^ dodine. Most actin patches show the characteristic dynamic behavior. Horizontal bar: 1 µm, vertical bar: 3 s. (**G**) Kymographs showing the dynamic behavior of actin patches in *U. maydis*, treated with the solvent methanol (Control) and with 20 µg ml^−1^ dodine. In the presence of dodine, F-actin patches are formed, but they do not enter phase 2 (”flickering“), nor disassemble (indicated by a continuous vertical line), suggesting that the endocytic vesicle formation at the PM is incomplete. Horizontal bar: 1 µm, vertical bar: 3 s. See also Video 4. (For interpretation of the references to colour in this figure legend, the reader is referred to the web version of this article.)

Fungal endocytosis is initiated by the assembly of “actin patches” at sites of endocytic vesicle formation (Kaksonen et al., 2006). A crucial step is the recruitment of actin and actin-associated proteins to the PM. Reorganization of actin and its polymerization exerts force that participates in formation of the endocytic vesicle, followed by propulsion of the cargo into the cytoplasm of the fungal cell (Kaksonen et al., 2003, Kaksonen et al., 2005, Liu et al., 2006, Munn, 2001). This release into the cytoplasm is indicated by motility of the actin patch (Kaksonen et al., 2003). To investigate the effect of dodine on this early endocytic step, we observed fluorescent actin patches in *Z. tritici* and *U. maydis*. We used the fluorescent probe Lifeact (Riedl et al., 2008), which we had previously adapted for use in the two fungi (Kilaru et al., 2017, Steinberg and Schuster, 2011). This probe labels F-actin cables and peripheral actin patches (Fig. 3A, Video 2; only *Z. tritici* is shown). Lifeact-labeled patches appeared at the PM and stand stationary, while increasing the amount of F-actin (Fig. 3B, 3C; “Assembly”; Video 3). This is followed by a plateau phase, where Lifeact fluorescence does not increase further, indicating that the endocytic vesicle is formed (Fig. 3B, 3C; “Internalization”). After scission of the vesicle, the Lifeact signal exhibits random motility and gradually disappears (Fig. 3B, 3C; “Random motility”; Video 3), suggesting that F-actin depolymerizes. This “three-phase” behavior was also found in *U. maydis*, but the average “life-time” of a patch (defined here as the time from the first appearance to the disappearance of the actin patch) differs significantly between both fungi (Fig. 3D; Student’s *t*-test; P < 0.0001).

We next investigated if the presence of dodine affects the formation and dynamic behavior of actin patches in *U. maydis* and *Z. tritici*. We tested the effect of the fungicide over a range of concentrations in the two fungi. In *Z. tritici*, actin patch showed the characteristic dynamic behavior in the presence of dodine, although the number of motile patches declined at high concentrations (Fig. 3E, 3F). In contrast, *U. maydis* actin patches were formed, but their dynamic behavior was strongly inhibited in the presence of the fungicide (Fig. 3E; 50% inhibition at EC_50_ = 5.95 µg ml^−1^), with almost no motility of actin patches at 20 µg ml^−1^ (Fig. 3E, 3G; compare to *Z. tritici* at same concentration in Fig. 3F; Video 4). These results strongly suggests that dodine-induced alterations of the *U. maydis* PM allows F-actin assembly at the site of endocytic uptake, but interferes with vesicle formation and/or internalization. In contrast, dodine has only mild effects on endocytic internalization at the PM in *Z. tritici*.

**Fig. 4:**
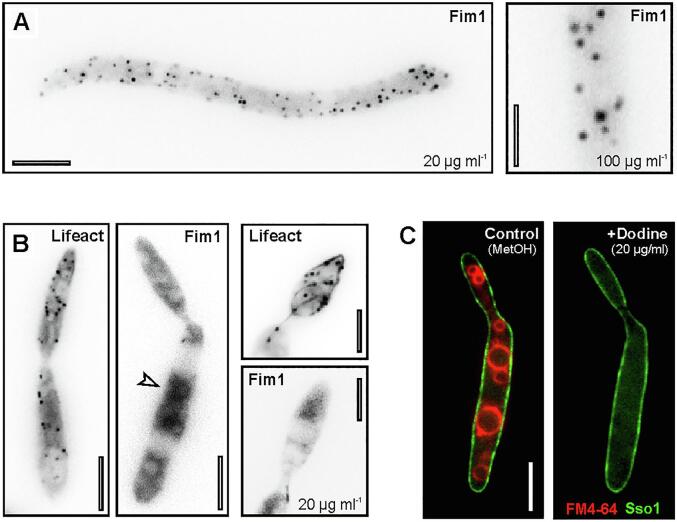
The effect of dodine on the recruitment of the actin crosslinking protein fimbrin. (**A**) Contrast-inverted image of GFP-Fim1-labelled actin patches in *Z. tritici* macropycnidiospores, treated with dodine at 20 µg ml^−1^ (left panel) and 100 µg ml^−1^ (right panel). Scale bars: 5 µm (left panel) and 2 µm (right panel). (**B**) Contrast-inverted image of Lifeact-labelled actin patches (Lifeact) and fluorescent fimbrin (Fim1) in *U. maydis* cells, treated with dodine at 20 µg ml^−1^. Note that actin concentrations are visible, but no specific and patchy fimbrin signal is seen. Scale bars: 5 µm (left panels) and 3 µm (right panel). (**C**) Pulse chase experiment, showing the delivery of the endocytosis reporter dye FM4-64 (red) to vacuoles in a *U. maydis* cell, treated with the solvent (Control) or 20 µg ml^−1^ dodine. The PM is labeled with GFP-Sso1 (green). Note that dodine treatment abolishes uptake of FM4-64, demonstrating that dodine inhibits endocytosis. Scale bar: 5 µm. (For interpretation of the references to colour in this figure legend, the reader is referred to the web version of this article.)

During actin patch formation, numerous actin-binding proteins are recruited that support action polymerization and reorganization during internalization of endocytic vesicles (Kaksonen et al., 2003, Kaksonen et al., 2005, Skau et al., 2011). Amongst these proteins is fimbrin, which localizes to fungal actin patches in yeasts (Huckaba et al., 2004, Wu et al., 2001) and filamentous fungi (Delgado-Alvarez et al., 2010, Upadhyay and Shaw, 2008), where it cross-links fungal F-actin (Adams et al., 1991, Skau et al., 2011, Wu et al., 2001). Studies in mutants in the S*. cerevisiae* fimbrin orthologue (Sac6p) demonstrated that fimbrin is not required for actin assembly at the PM, but is crucial for patch motility (Kaksonen et al., 2005). This phenotype is reminiscent of the effect of dodine on actin patch motility in our experiments. Thus, we considered it possible that fungicide-treated cells do not recruit fimbrin to their actin patches. We reported previously the visualization of fimbrin in actin patches in *U. maydis* (Castillo-Lluva et al., 2007) and *Z. tritici* (Kilaru et al., 2015). We used these GFP-Fim1 expressing strains and treated them with dodine. In *Z. tritici* cells, GFP-Fim1 signals were still located at the cell periphery (Fig. 4A; 20 µg ml^−1^ and 100 µg ml^−1^ dodine for 30 min is shown), and showed the same dynamic behavior as Lifeact-labelled actin patches (not shown). However, in *U. maydis* cells that were treated with 20 µg ml^−1^ dodine no peripheral fimbrin signals were seen (Fig. 4B; Fim1), while stationary Lifeact-labelled F-actin patches were visible (Fig. 4B; Lifeact). Instead, the GFP-fusion protein accumulated in the cytoplasm of the cells (Fig. 4B, arrowhead), suggesting that dodine prevents the recruitment of fimbrin to actin patches thereby preventing endocytic vesicle internalization, which is expected to inhibit endocytosis. We tested this further in pulse-chase experiments, using the endocytic marker dye FM4-64, which is taken up into the cell by endocytosis and accumulates in the vacuolar tonoplast (Fischer-Parton et al., 2000, Wedlich-Söldner et al., 2000). Indeed, at 45 min after applying the dye, FM4-64 concentrated in the membrane of rounded vacuoles in control cells (Fig. 4C, left panel). However, cells that were pre-treated with 20 µg ml^−1^ dodine did not deliver FM4-64 into the vacuoles (Fig. 4C, right panel). This finding is consistent with the observed dodine-induced arrest in endocytic uptake at the PM. Thus we conclude that dodine inhibits endocytosis in *U. maydis*, which represents a novel MoA of this fungicide.

**Fig. 5:**
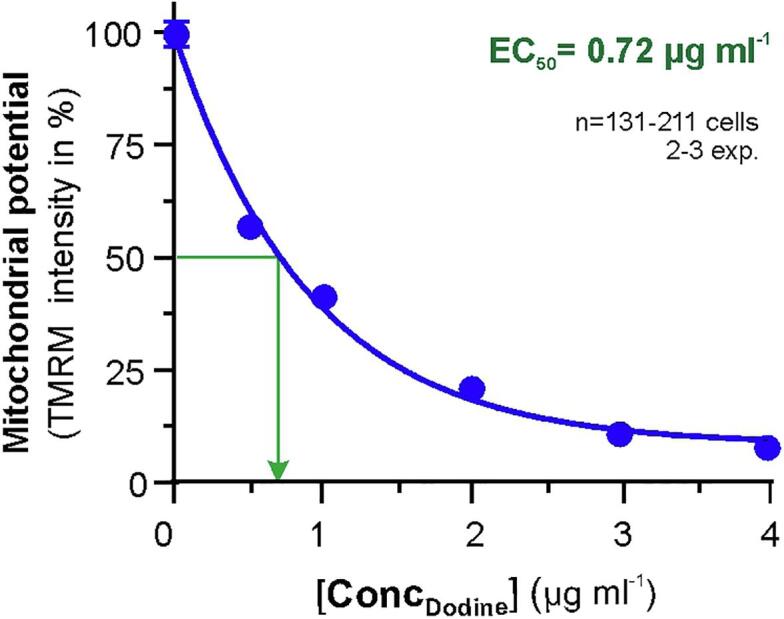
The effect of dodine at various concentrations on the mitochondrial membrane potential in *U. maydis*, indicated by TMRM fluorescence intensity. Cells were incubated for 30 min. Data points are given as mean ± standard error of the mean (SEM) from 131 to 211 cells from 2 to 3 experiments. Concentration of dodine at 50% inhibition of the mitochondrial inner membrane potential is indicated by EC_50_ value (green arrow; determined graphically from a non-linear regression curve, calculated as dose response inhibition, four parameters, in Prism6). (For interpretation of the references to colour in this figure legend, the reader is referred to the web version of this article.)

Endocytosis was shown to be important for morphogenesis and pathogenicity of *U. maydis* (Fuchs et al., 2006, Wedlich-Söldner et al., 2000). We demonstrate here that endocytic internalization is inhibited at low concentrations of dodine (EC_50_ = 5.95 µg ml^−1^; Fig. 3E). This raises the possibility that this MoA is responsible for the lethal activity of this fungicide on the corn smut fungus, a conclusion previously drawn by others (Solel and Siegel, 1984). However, growth of *U. maydis* on agar plates is inhibited at lower dodine concentrations (EC_50_ = 2.29 µg ml^−1^; Steinberg et al., 2020). This raises doubt about the effect at the PM being the primary reason for its fungicidal activity. Indeed, dodine, as well as other mono-alkyl lipophilic cations, have recently been shown to interfere with mitochondrial respiration and oxidative phosphorylation in *U. maydis* (Steinberg et al., 2020). However, in comparing these EC_50_ values, one must be mindful that endocytic uptake inhibition was measured after 30 min in liquid culture, whereas growth inhibition is detected after several days on solid media. To allow a more direct comparison between the effect on mitochondria and the plasma membrane, we tested various dodine concentrations on the *U. maydis* mitochondrial membrane potential, using staining of the mitochondria with tetramethylrhodamine methyl ester (TMRM, Scaduto and Grotyohann, 1999). These experiments revealed that inhibition of the mitochondrial membrane potential occurs at ∼ 8-times lower concentrations than needed to inhibit endocytosis (Fig. 5; EC_50_ = 0.72 µg ml^−1^). Such effect at low dodine concentrations was also found in *Z. tritici* (EC_50_ = 0.28 µg ml^−1^; Steinberg et al., 2020). The proton-gradient over the inner mitochondrial membrane provides the “proton-motive force” to synthesize ATP (Mitchell, 1961). We therefore consider it likely that the primary MoA of dodine in *U. maydis*, as well as in other fungi, is the inhibition of oxidative phosphorylation, and thereby ATP synthesis. However, dodine-induced inhibition of endocytosis and PM depolarization most likely increase the toxic effect of the fungicide in this pathogen.

In this study, we use cell biology techniques to investigate the MoA of dodine in living cells. Dodine is a well-established protectant fungicide, used to control apple scab and other foliar diseases (EPA 2005; https://archive.epa.gov/pesticides/reregistration/web/pdf/dodine-red.pdf), but its MoA is unknown (FRAC code© list 2020, classification U12; https://www.frac.info). We report here that dodine acts at the PM of *U. maydis*, where the fungicide has multiple effects: it (i) inserts in the membrane and causes PM invaginations, (ii) reduces the fluidity of the PM, (iii) alters the ion permeability, thereby depolarizing the cell and (iv) blocks endocytosis by arresting actin patches at sites of endocytic internalization. We found that the plasma membrane of *Z. tritici* reacts differently to dodine, with no obvious defect in its fluidity or integrity, and only mild effects on actin patch formation. Plasma membrane invaginations, which most likely indicate considerable insertions of dodine, occur at lower concentrations in *U. maydis* than in *Z. tritici*. Thus, we consider it possible that the lipophilic cation inserts more easily into the *U. maydis* plasma membrane. Alternatively, the cationic dodine may be held longer in the basidiomycete plasma membrane before it diffuses into the cell. Such extended residency would increase the dodine concentration in the membrane and so underpin the difference between both fungi. But why does the plasma membranes of both fungi show differential affinity for dodine? The answer may lie in their phylogenetic relationship. *U. maydis* belongs to the phyllum basidiomycota (Ustilaginomycotina), whereas *Z. tritici* is an ascomycete (Pezizomycotina). Both phyla diverged ∼ 500 million years ago (Stajich et al., 2009), suggesting independent evolution of certain cellular processes. Indeed, *U. maydis* contains numerous proteins and cellular pathways, relevant for human diseases, that are absent from the ascomycetes (Münsterkötter and Steinberg, 2007; Steinberg and Perez-Martin, 2008). It is therefore likely that the plasma membrane composition differs between basidio- and ascomycete fungi. Indeed, the lipid and protein content of the plasma membrane of *U. maydis* and the ascomycete *Penicillium cyclopium* show significant differences in lipid abundance between both fungi (Supplementary Fig. 3; Hernández et al., 1994). Moreover, a comparative analysis of the polar lipid content in a filamentous ascomycete (*Neurospora crassa*)*,* and two basidiomycetes (*Schizophyllum commune*, *Polyporus versicolor*) reveals that the basidiomycete fungi contain ∼ 2.1–2.6-times more phosphotidylserine in their cell membranes (Hendrix and Rouser, 1976; Supplementary Fig. 3). How such differences affect the integration of dodine into the plasma membrane is unclear. One possibility is that the negative head groups of phosphotidylserines capture the positively-charged dodine molecule, thereby increasing the fungicide content in the plasma membrane of the basidiomycete *U. maydis*. We show that this insertion changes membrane fluidity, a parameter that is dependent on the lipid composition of the fungal membrane (Florek et al., 2018). Moreover, enrichment of dodine also increases positive charges on the plasma membrane surfaces. This is likely affects voltage-gated transmembrane channels (Catterall, 2010), which may cause the observed depolarization of the plasma membrane in *U. maydis*.

**Fig. 6:**
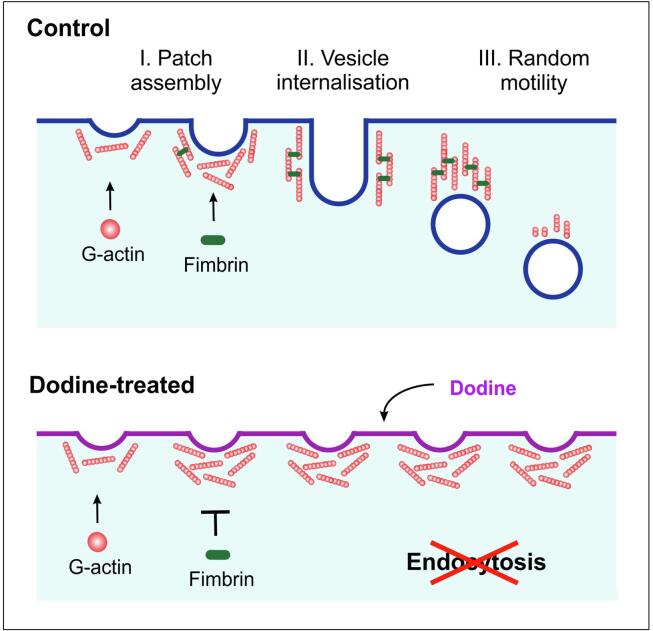
Model of the effect of early endocytic steps in *U. maydis*. During patch assembly, G-actin gets recruited, which polymerizes into F-actin. Amongst other patch proteins, the cross-linking protein Fim1 is incorporated into the assembling actin patch, which allows actin polymerization to exert force, required for vesicle formation and internalization. After scission, polymerization of bundled actin moves vesicles away from the PM, which is followed by depolymerization of F-actin. In the presence of dodine in the PM, fibrin is not recruited to the actin patch. Consequently, actin is not cross-liked and force cannot be exerted, which is indicated by the absence of random patch motility. The consequence is a block of endocytic internalization at the PM.

We also show that endocytic vesicle formation is impaired in dodine-treated *U. maydis* cells. In the yeast *S. cerevisiae* the lipid composition of the plasma membrane controls endocytic uptake (Munn et al., 1999), suggesting that altered membrane fluidity could underpin the endocytosis defect. Our data demonstrate that fimbrin is not recruited to the site of endocytic internalization, which coincides with abolished actin patch dynamics. Fimbrin mutants in *S. cerevisiae* show a block in actin patch dynamics (Kaksonen et al., 2005). We therefore consider it possible that a defect in targeting fimbrin to the PM is sufficient to explain the inhibition of endocytic internalization in dodine-treated cell of *U. maydis* (Fig. 6). This crosslinker directly binds F-actin (Klein et al., 2004), suggesting that actin polymerization recruits fimbrin. However, our results show that F-actin accumulates at the plasma membrane in dodine-treated cells. Thus, the obvious next question is, why is the actin-binding protein fimbrin not recruited to these immature actin patches? We have no definitive answer to this question. However, it is worth noting that fimbrin activity is regulated by an N-terminal domain that contains 2 EF-hand modules, implying calcium regulation (Klein et al., 2004). Work in the fission yeast *Schizosaccharomyces pombe* confirmed the importance of the N-terminal region (Nakano et al., 2001), but also showed that calcium is not involved in the actin-binding or cross-linking activity of fimbrin (Nakano et al., 2001). This, suggests that the N-terminal region interacts with other factors, which may control recruitment of fimbrin to F-actin. Interestingly, in mammalian cells the protein Iba1 binds to fimbrin and enhances its actin-bundling activity (Ohsawa et al., 2004). While such a fimbrin recruiting factor has not been identified in fungi, these results demonstrate that fimbrin recruitment to F-actin patches could depend on the molecular environment of the actin patch. This notion is further supported by recent findings that other actin-binding proteins compete with fimbrin for interaction with F-actin (Christensen et al., 2019). Actin patches contain > 50 different proteins (Goode et al., 2015), and we consider it possible that introduction of dodine-associated cationic charges into the plasma membrane could perturb the fine balance between these actin patch proteins, thereby interfering with fimbrin recruitment and activity.

In this paper, we show a differential effect of dodine on the plasma membrane of the basidiomycete fungus *U. maydis* and the ascomycete fungus *Z. tritici*. While the fungicidal effect of dodine in *Z. tritici* is due to inhibition of mitochondrial respiration, which results in ATP depletion (Steinberg et al., 2020), the fungicide has a broader MoA in *U. maydis*. Here, dodine inhibits oxidative phosphorylation, but, in addition, appears to concentrate in the plasma membrane, where it (i) compromises the permeability of the plasma membrane to ions and (ii) inhibits endocytosis. However, the effects at the plasma membrane occur at much higher concentrations (EC_50, respiration_: 26.5 µg ml^−1^, EC_50, endocytosis_ = 5.95 µg ml^−1^, EC_50, mitochondrial potential_ = 0.72 µg ml^−1^; Fig. 3E; Fig. 5; Supplementary Fig. 2). Thus we conclude that the inhibition of oxidative phosphorylation is also the primary MoA in *U. maydis.* These results emphasize that the (i) MoA can differ between fungi, in particular if they are phylogenetically distant, and (ii) quantitative and standardized studies are required to distinguish between primary and secondary effects. Clearly, the analysis of fungicide effects in living pathogen cells provides a unique and powerful approach to the study of the MoA of a given fungicide.

## 3 Methods

### 3.1 Strains used in this study

The *U. maydis* strains FB1, FB1GSso1, AB33GLifeact and FB2Fim2G were published previously (Banuett and Herskowitz, 1989, Steinberg and Schuster, 2011, Steinberg et al., 2020, Theisen et al., 2008). The *Z. tritici* strains IPO323, IPO323_eGFP-Sso1, IPO323_Lifeact-ZtGFP and IPO323_HFim1eGFP were also published elsewhere (Kema and van Silfhout, 1997, Kilaru et al., 2015, Kilaru et al., 2017). Lists of strain genotypes and their experimental usage are provided in Supplementary Table 1 and Supplementary Table 2.

### 3.2 Culture conditions

Long-term storage of all fungal strains was at −80 °C in NSY glycerol (nutrient broth, 8 g l^−1^; yeast extract, 1 g l^−1^; sucrose, 5 g l^−1^; glycerol, 700 ml l^−1^, Sigma Aldrich, Poole UK). For daily use, *U. maydis* strains were maintained on agar plates (1% agar (w v^−1^), 1% glucose (w v^−1^) in complete medium; CM, Holliday, 1974; see Supplementary Information for detailed recipe), from where liquid cultures were grown in complete medium, supplemented with 1% (w v^−1^) glucose (CM_glucose_), at 28 °C for 12 h, 200 rpm. Frozen *Z. tritici* NSY glycerol stocks were streaked onto YPD agar plates (yeast extract, 10 g l^−1^; peptone, 20 g l^−1^; glucose, 20 g l^−1^; agar, 20 g l^−1^, Sigma Aldrich) and grown at 18 °C for 5 days. From here, YG cultures (yeast extract, 10 g l^−1^; glucose, 30 g l^−1^) were inoculated and grown at 18 °C for 48 h, shaking at 200 rpm.

### 3.3 Live cell imaging

Cells were placed onto 2% (w v^−1^) agar cushions and observed using an IX83 motorized inverted microscope (Olympus, Hamburg, Germany), equipped with a PlanApo × 100/1.45 oil TIRF objective (Olympus) and a VS-LMS4 Laser-Merge-System with solid-state lasers (488 nm/ 70 mW and 561 nm/ 70 mW, Visitron System, Puchheim, Germany). For photo-bleaching experiments, a 405 nm/ 60 mW diode laser was used, which was coupled into the light path by an OSI-IX 71 adaptor (Visitron System) and controlled by a UGA-40 controller (Rapp OptoElectronic, Hamburg, Germany) and VisiFRAP 2D FRAP control software. Z stacks were generated by using an objective piezo (Piezosystem Jena GmbH, Jena, Germany). Images were acquired using a Photometrics CoolSNAP HQ2 camera (Photometrics/ Roper Scientific, Tucson, USA). All parts of the system were under the control of the software package VisiView (Visitron System). Samples were observed for no longer than 10 min, to prevent oxygen depletion. All image processing was performed in MetaMorph 7.8.x (Molecular Devices, Wokingham, UK).

### 3.4 Cell staining methods

For all experiments, one milliliter of overnight cell cultures were treated with different concentrations of dodine (Sigma Aldrich; stock solution: 10–100 mg ml^−1^ in methanol) or the respective amount of methanol alone (solvent control), for 30–45 min at room temperature, rotating on a SB2 Rotator (Bibby Scientific, Limited, Stone, UK).

*Plasma membrane potential*: To investigate the effect of dodine-treatment on the permeability of the PM for ions, cells were incubated for 5 min with the voltage-sensitive fluorescent dye DiBAC_4_(3) (bis-(1,3-dibutylbarbituric acid)tri, methine oxonol; Thermo Fisher Scientific, Loughborough, UK; stock: 1 mg ml^−1^) at a final concentration 20 µg ml^−1^. Subsequently, cells were washed twice with CM or YG media by centrifugation using a Micro Star 17R cooled centrifuge (VWR, Lutterworth, UK) at 3500 or 5000 rpm for 5 min, followed by immediate microscopic analysis using the 488 nm laser at 20% intensity and exposure time of 150 ms. The total number of cells and the number of stained cells was recorded and the percentage of stained cells was determined. The mean ± SEM from 3 experiments was calculated using Prism 5.

*Cell survival*: To assess cell death, 100 μl of dodine-treated cell cultures were incubated with 1 µl propidium iodide (Sigma Aldrich; stock: 1 mg ml^−1^ in ddH_2_O). After 5 min incubation at room temperature, 1 µl of cell suspension was placed onto a 2% (w v^−1^) agar cushion and analyzed using the 488 nm and 561 nm lasers, both set at 20% intensity and exposure time of 150 ms. The total number of cells and the number of stained cells was recorded and the percentage of stained cells was determined. The mean ± SEM from 3 experiments was calculated using Prism 5.

*Endocytic vacuolar sorting:* To investigate the effect of dodine on endocytic uptake into the vacuole, 1 ml of the dodine-treated *U. maydis* cells was incubated with 1 μl of the endocytosis reporter dye FM4-64 (Thermo Fisher Scientific; stock 16 mM in DMSO) for 5 min in the dark at room temperature. Cells were washed twice with CM media by centrifugation using a Micro Star 17R cooled centrifuge (VWR, Lutterworth, UK) at 3500 rpm for 5 min. This was followed by resuspension of the cell pellet 1 ml CM_glucose_ and incubation on a SB2 Rotator in the dark at room temperature for 45 min. 1 µl cell suspension was placed onto a 2% (w v^−1^) agar cushion and microscopically images were taken (488 nm and 561 nm lasers at 20% or 50% output power and an image exposure time of 150 ms.

*Mitochondrial membrane potential*: Polarization of mitochondria was visualized using tetramethylrhodamine methyl ester (TMRM; Thermo Fisher Scientific) as described previously (Steinberg et al., 2020). In brief, 1 ml of cell culture, grown in CM_glucose_, was treated with various concentrations of dodine or the solvent methanol and incubated for 30 min at room temperature, rotating on a SB2 Rotator. Subsequently 1 μl TMRM was added, and the mixture was incubated in the dark at room temperature for 10 min, rotating on a SB2 Rotator. 1 µl cell culture was placed onto a 2% (w v^−1^) agar cushion and TMRM was imaged using 561 nm laser, at 20% intensity, whereas the GFP-Sso1-carrying PM was visualized with a 488 nm laser at 20% intensity and an exposure time of 150 ms. To quantitatively determine the mitochondrial potential, a region of interest, covering the entire cell within the confinement of the PM, labelled with GFP-Sso1, was generated. This region was transferred to the image of TMRM fluorescence and the integrated signal intensity was measured. The value was corrected by the integrated signal intensity within the same region in the image background. The mean ± SEM from 131 to 211 measurements, done in 2–3 experiments was plotted in Prism 6.

### 3.5 Analysis of membrane appearance

The effect of dodine on plasma membrane appearance was analyzed in strains FB1GSso1 and IPO323_eGFP-Sso1. Treated cells were paced onto a 2% (w v^−1^) agar cushion and z-stacks were taken at a z step size of 0.2 μm with the 488 nm laser at 20% and an exposure time of 150 ms. The total number of cells and the number cells showing PM alterations was recorded and the percentage of stained cells was calculated and a graph with the mean of 3 experiments was generated.

### 3.6 FRAP experiments to investigate PM fluidity

The effect of dodine treatment on the fluidity of the fungal PM fluidity was investigated by fluorescent recovery experiments. To this end, 1 ml of overnight *U. maydis* and *Z. tritici* cultures were treated with various concentrations of dodine or the solvent control, containing equivalent amounts of methanol, for 30 min at room temperature, rotating on a SB2 Rotator. 1 μl of the cell suspensions was placed onto a 2% (w v^−1^) agar cushion and a reference image was taken. A central area of 2–3 μm was photo bleached, using a 405 nm laser at 80% output power, followed by immediate image acquisition at 30 sec intervals for 5 min. Fluorescence recovery was measured as average intensity in the bleached area of the PM using MetaMorph. In parallel, the signal intensity in an unbleached area was measured. All fluorescent intensity values were corrected for adjacent image background, and signal intensities in photo-bleached regions were compared to those in unbleached parts of the PM.

### 3.7 Effect of dodine on actin patch dynamics

The effect of dodine treatment on actin patch dynamics was visualized in strains AB33GLifeact and IPO323_Lifeact-ZtGFP. To this end, 1 ml of overnight *U. maydis* and *Z. tritici* cultures were treated with various concentrations of dodine or the solvent control, containing equivalent amounts of methanol, for 30 min at room temperature, rotating on a SB2 Rotator. 1 μl of the cell suspensions was placed onto a 2% (w v^−1^) agar cushion and movies of 100–150 plains with an exposure time of 150 ms and the 488 nm laser at 10% was acquired. From those movies, kymographs of single cells were generated. The total number of actin patches and the number of actin patches showing normal dynamic behavior was recorded and the percentage of dynamic patches was calculated. Only patches which showed a full circle of assembly, plateau and scission within 15 s were considered as dynamic. Patches which were visible in plane 1 and did not show any sign of scission within 15 s were considered as non-dynamic. Patched which got assembled within the 15 s observation window but plateaued till the end of the 15 s were not considered at all.

### 3.8 Image processing and statistical analysis

All images were adjusted for brightness, contrast and gamma values using MetaMorph 7.8.x or Adobe Photoshop CS6. All statistical were done using the software GraphPad Prism 5 or 6 (GraphPad, San Diego, USA). Data sets were tested firstly for normal distribution using the Shapiro-Wilk test for normality. In case only two data sets were compared, all data sets which passed that test were statistically compared using a Student’s *t*-test with Welch’s correction. Data sets that did not passed a Shapiro-Wilk test for normality were compered using a nonparametric two-tailed Mann-Whitney test. If more than two data sets were statistically compared a one-sided ANOVA test was used for normal distributed data sets. All data sets which did not passed a Shapiro-Wilk test for normality (P > 0.05) were compared using a Kruskal-Wallis test. Non-linear regression curves were calculated as dose response inhibition (four parameters) in Prism6; EC_50_ values were determined graphically.

### 3.9 Electron microscopy

Transmission electron microscopy in *U. maydis* cells, treated with 50 µg ml^−1^ dodine, was done as described (Schuster et al., 2016, Steinberg et al., 2017b) using a JEOL JEM 1400 transmission electron microscope, operated at 120 kV. Image acquisition was done using a digital camera (ES 1000 W, Gatan, Abingdon, UK). Cells were centrifuged at 4,000 g for 10 min, and the cell sediment was fixed in 2% (v v^−1^) glutaraldehyde and 2% (v v^−1^) formaldehyde in 0.1 M PIPES buffer pH 7.2. Samples were post-fixed with 2% (w v^−1^) potassium permanganate in dH_2_O, then dehydrated and embedded in Durcupan resin (Sigma-Aldrich). 60 nm ultrathin sections were collected on pioloform-coated 100-mesh copper EM grids (Agar Scientific, Stanated, UK), contrasted with lead citrate and analyzed using the JEOL JEM 1400 transmission electron microscope.

## CRediT authorship contribution statement

**Martin Schuster:** Data curation, Investigation, Formal analysis. **Gero Steinberg:** Conceptualization, Visualization, Formal analysis, Writing - review & editing, Supervision, Project administration, Funding acquisition.
