# Supplementary material for: The fungicide dodine primarily inhibits mitochondrial respiration in Ustilago maydis, but also affects plasma membrane integrity and endocytosis, which is not found in Zymoseptoria tritici
Source: Fungal Genet Biol. 2020 Sep;142:103414. doi: 10.1016/j.fgb.2020.103414 (PMC7526662; doi:10.1016/j.fgb.2020.103414)
Supplement: Supplementary data 1 [file mmc1.docx]

**Supplementary Material**

**Supplementary Figures**

**
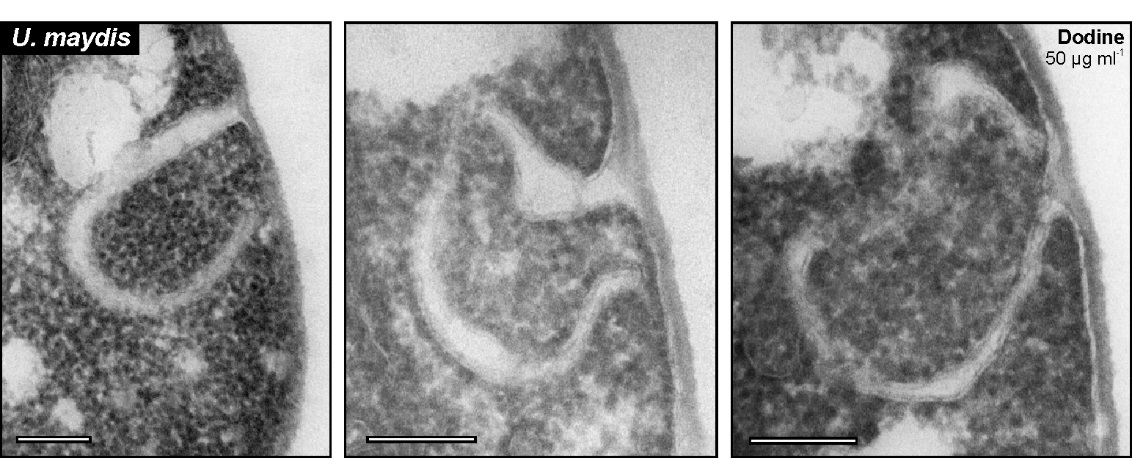
**

**Supplementary Figure 1**

Ultrastructure of PM invaginations in *U. maydis* cells, treated with 50 µg ml^-1^ dodine. Scale bars represent 0.2 µm.


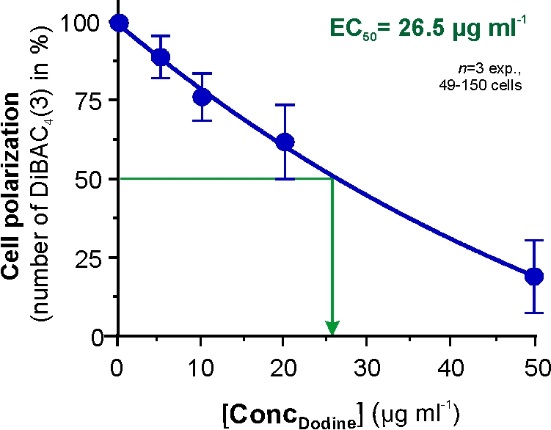


**Supplementary Figure 2**

Graph showing the effect of various concentrations of dodine on the depolarization of *U. maydis* cells, determined by the number of DiBAC_4_(3)-positive cells. Cells were incubated for 30 minutes. Data points are given as mean ± standard error of the mean (SEM) from 49-150 cells from 3 experiments. Concentration of dodine at 50% depolarization of *U. maydis* cells is indicated by EC_50_ value (determined graphically from a non-linear regression curve, calculated as dose response inhibition (four parameters) in Prism6; green arrow).


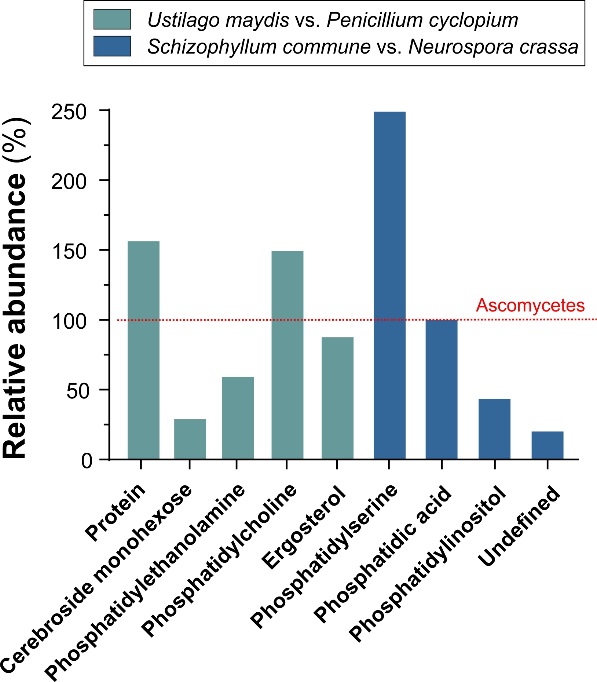


**Supplementary Figure 3**

Comparison of plasma membrane proteins and membrane lipids in basidiomycetes and ascomycete. The measurements for the ascomycete fungi (*N. crassa* to compare to blue bars that represent *Schizophyllum commune*; *Penecillium cyclopium* to compare to green-grey bars that represent *Ustilago maydis*) were set to 100% and are indicated by a red-dotted line. Note the dominance of negatively charged phosphatidylserines in basidiomycete membranes. Data were taken from Hendrix and Rouser, 1976 (blue bars and corresponding ascomycete) and Hernández et al. 1994 (grey bars and corresponding ascomycete).

| **Supplementary Table 1** Fungal strains and plasmids used in this study | | | |
| --- | --- | --- | --- |
| **Strain name** | **Genotype** | **Reference** |  |
| ***Z. tritici*** |  |  |  |
| IPO323 | wildtype, *MAT1-1* | (Kema and van Silfhout, 1997) |  |
| IPO323_eGFP-Sso1 | *MAT1-1* / pCeGFPSso1 | (Kilaru et al., 2017) |  |
| IP0323_ Lifeact-ZtGFP | *MAT1-1* / pCLifeactZtGFP | (Kilaru et al., 2017) |  |
| IPO323_HFim1eGFP | *MAT1-1* / pCFim1eGFP | (Kilaru et al., 2015) |  |
|  |  |  |  |
| ***U. maydis*** |  |  |  |
| FB1 | wildtype*, a1 b1* | (Banuett and Herskowitz, 1989) |  |
| FB1GSso1 | *a1 b1* / poGSso1 | (Steinberg et al., 2020) |  |
| AB33GLifeact | *a2* P*narbW2* P*narbE1, ble*^R^*/* poGLifeact | (Steinberg and Schuster, 2011) |  |
| FB2Fim2G | *a2b2 fim2-gfp, ble*^R^ | (Theisen et al., 2008) |  |
| **Plasmid name** | **Genotype** | **Reference** |  |
| pCeGFPSso1 | P*tub1-eGFP-sso1*, *cbx*^R^ | (Kilaru et al., 2017) |  |
| pCLifeactZtGFP | P*tub1-Lifeact-ZtGFP*, *cbx*^R^ | (Kilaru et al., 2017) |  |
| pCFim1eGFP | P*tub1-fim1-eGFP*, *cbx*^R^ | (Kilaru et al., 2015) |  |
| poGSso1 | P*otef-egfp-sso*1, *cbx*^R^ | (Steinberg and Schuster, 2011) |  |
| poGLifeact | P*otef-egfp-lifeact*, *cbx*^R^ | (Steinberg and Schuster, 2011) |  |
| *cbx*^R^ or C, carboxin resistance; *ble*^R^ *,* phleomycin resistance; *a*, *b*, mating type loci; *nar*, conditional nitrate reductase promoter; E1, W2, genes of the b mating type locus; *egfp*, enhanced green fluorescent protein; *Ztgfp*, *Z. tritici* codon-optimized enhanced green fluorescent protein; *sso*1, a syntaxin-like plasma membrane protein; Lifeact, first 17 aa of the actin-binding protein Abp140; fim1, actin-crosslinking protein fimbrin; MAT, mating type; P*otef*, constitutive promoter; P*tub1*, αtubulin promoter | | | |

| **Supplementary Table 2** Experimental usage of fungal strains | | | |
| --- | --- | --- | --- |
| **Strain name** | **Type of experiment** | **Reference** | |
| ***Z. tritici*** |  | |  |
| IPO323 | Live/Dead staining; PM permeability | | Fig. 1f-h |
| IPO323_eGFP-Sso1 | Membrane appearance/fluidity | | Fig. 1b, 2c, 2d |
| IP0323_ Lifeact-ZtGFP | Visualize actin patches and analyzse dynamics | | Fig. 3a-e, g |
| IPO323_HFim1eGFP | Visualise the fimbrin in actin patches | | Fig. 4a |
| ***U. maydis*** |  | |  |
| FB1 | Live/Dead staining; PM permeability | | Fig. 1c-e; S2 |
| FB1GSso1 | Membrane appearance and fluidity; endocytic vacuolar sorting; electron microscopy; mitochondrial potential | | Fig. 1a, 1b, 2a, 2b; 4c, S1; |
| AB33GLifeact | Analyses of actin patches dynamics | | Fig. 3d, 3f, 3g |
| FB2Fim2G | Visualise fimbrin in actin patches | | Fig. 4b |

**Supplementary Methods**

**Composition of complete medium/glucose (CM_glucose_) for *U. maydis* liquid cultures** (Holliday 1974)

2.5 g l^-1^ casamino acids; 1 g l^-1^ yeast extract; 10 ml l^-1^; 0.5 g l^-1^ deoxyribonucleic acid from herring sperm; 1.5 g l^-1^ NH_4_NO_3_; 1% (w v^-1^) glucose; 10 ml l^-1^ vitamin solution, 62.5 ml l^-1^ salt solution; 8 ml l^-1^

The vitamin solution: 100 mg l^-1^ thiamine hydrochloride; 50 mg l^-1^ riboflavin; 50 mg l^-1^ pyridoxine hydrochloride; 200 mg l^-1^ D-pantothenic acid hemicalcium salt ; 50 mg l^-1^4-Aminobenzoic acid; 200 mg l^-1^ nicotinic acid; 200 mg l^-1^ choline chloride; 1 g l^-1^ *myo*-Inositol.

The salt solution: 16 g l^-1^ KH_2_PO_4_; 4 g l^-1^ Na_2_SO_4_; 8 g l^-1^ KCl; 4.08 g l^-1^ MgSO_4_*7H_2_O; 1.32 g l^-1^CaCl_2_*2H_2_O

Trace elements solution: 60ml l^-1^ H_3_BO_3_; 140 mg l^-1^ MnCl*4H_2_O; 400 mg l^-1^ ZnCl_2_; 40 ml l^-1^ Na_2_MoO_4_*2H_2_O; 100 mg l^-1^ FeCl_3_*6H_2_O; 40 mg l^-1^CuSO_4_*5H_2_O.

All ingredients from Sigma Aldrich (https://www.sigmaaldrich.com/catalog/search).

**Supplementary Video legends**

**Video 1** The effect of dodine on plasma membrane fluidity in *U. maydis*. Movie shows the recovery of fluorescence of plasma membrane-bound syntaxin GFP-Sso1 in *U. maydis* cells, treated with the solvent methanol (Control) and 10 µg ml^-1^ dodine. Time is given in the lower right corner. The photo-bleached area is indicated by a box and “Laser” in the second frame. Scale bar represents 3 µm.

**Video 2** F-actin in the tip of a macropycnidiospore of *Z. tritici*. Actin cables and actin patches are indicated. All structures are labelled with the fluorescent marker lifeact, which only binds to actin in its polymerized form (F-actin). Note that F-actin is predominantly located at the cell periphery. Scale bar represents 3 µm.

**Video 3** Dynamic behavior of peripheral actin patches in *Z. tritici*. Actin structures are labelled with the fluorescent marker Lifeact. Note that stationary patches appear at random sites, followed by an increase in fluorescent intensity, before they go into random motility and disappear. Time is given in seconds and milliseconds in the lower right corner. Scale bar represents 1 µm.

**Video 4** Effect of dodine on actin patch dynamics in *U. maydis*. Upper panel shows Lifeact-labelled patches in cells incubated with the solvent methanol (Control). The lower panel shows actin patches in cells that were treated with 20 µg ml^-1^ dodine for 30 minutes. Note that F-actin still accumulates in patches (arrowhead), but these patch remain stationary, suggesting that endocytic internalization is arrested. Time is given in seconds and milliseconds in the lower right corner Scale bar represents 5 µm.

**Supplementary References**

Banuett, F., Herskowitz, I., 1989. Different *a* alleles of *Ustilago maydis* are necessary for maintenance of filamentous growth but not for meiosis. Proc Natl Acad Sci U S A. 86, 5878-5882

Hendrix, J.W., G. Rouser, G. 1976. Polar lipids of *Phytophthora parasitica* var. *nicotianae*, in comparison with those of selected other fungi. Mycologia. 68, 354-361.

Hernández, A., Cooke, D.T., Clarkson, D.T. 1994. Lipid composition and proton transport in *Penicillium cyclopium* and *Ustilago maydis* plasma membrane vesicles isolated by two-phase partitioning. Biochim. Biophys. Acta 1195,103-109.

Kema, G.H.J., and C.H. van Silfhout. 1997. Genetic variation for virulence and resistance in the wheat-*Mycosphaerella graminicola* pathosystem. III. Comparative seedling and adult plant experiments. *.* Phytopathol. 87, 266–272.

Kilaru, S., Schuster, M., Latz, M., Guo, M., Steinberg, G., 2015. Fluorescent markers of the endocytic pathway in *Zymoseptoria tritici*. Fungal Genet. Biol. 79, 150-157.

Kilaru, S., Schuster, M., Ma, W., Steinberg, G., 2017. Fluorescent markers of various organelles in the wheat pathogen *Zymoseptoria tritici*. Fungal Genet. Biol. 105, 16-27.

Steinberg, G., Schuster, M., 2011. The dynamic fungal cell. Fungal Biol. Rev. 25, 14-37.

Steinberg, G., Schuster, M., Gurr, S.J., Schrader, T., Schrader, M., Wood, M., Early, A., Kilaru, S., 2020. A lipophilic cation protects crops against fungal pathogens by multiple modes of action. Nat. Commun. 11, 1608.

Theisen, U., Straube, A., Steinberg, G., 2008. Dynamic rearrangement of nucleoporins during fungal "open" mitosis. Mol. Biol. Cell. 19,1230-1240.
